# Non-arbitrary mappings between size and sound of English words: Form typicality effects during lexical access and memory

**DOI:** 10.1177/17470218231184940

**Published:** 2023-07-06

**Authors:** Greig I de Zubicaray, Joanne Arciuli, Frank H Guenther, Katie L McMahon, Elaine Kearney

**Affiliations:** 1 School of Psychology and Counselling, Faculty of Health, Queensland University of Technology, Brisbane, QLD, Australia; 2 College of Nursing and Health Sciences, Flinders University, Adelaide, SA, Australia; 3 Department of Speech, Language & Hearing Sciences, Boston University, Boston, MA, USA; 4 Department of Biomedical Engineering, Boston University, Boston, MA, USA; 5 School of Clinical Sciences, Centre for Biomedical Technologies, Queensland University of Technology, Brisbane, QLD, Australia; 6 Herston Imaging Research Facility, Royal Brisbane and Women’s Hospital, Herston, QLD, Australia

**Keywords:** Form typicality, megastudy, semantic size, embodiment, word recognition, memory

## Abstract

A century of research has provided evidence of limited size sound symbolism in English, that is, certain vowels are non-arbitrarily associated with words denoting small versus large referents (e.g., /i/ as in *teensy* and /ɑ/ as in *tall*). In the present study, we investigated more extensive statistical regularities between surface form properties of English words and ratings of their semantic size, that is, *form typicality*, and its impact on language and memory processing. Our findings provide the first evidence of significant word form typicality for semantic size. In five empirical studies using behavioural megastudy data sets of performance on written and auditory lexical decision, reading aloud, semantic decision, and recognition memory tasks, we show that form typicality for size is a stronger and more consistent predictor of lexical access during word comprehension and production than semantic size, in addition to playing a significant role in verbal memory. The empirical results demonstrate that statistical information about non-arbitrary form-size mappings is accessed automatically during language and verbal memory processing, unlike semantic size that is largely dependent on task contexts that explicitly require participants to access size knowledge. We discuss how a priori knowledge about non-arbitrary form-meaning associations in the lexicon might be incorporated in models of language processing that implement Bayesian statistical inference.

The notion that words’ meanings are represented via mental simulation, or automatic reactivation of a sensory experience of a word’s referent, is central to accounts of grounded cognition (Barsalou, 2008). Multiple empirical studies have therefore attempted to provide evidence for simulation of object percepts, such as size, shape, and orientation during various language and memory tasks (for a meta-analysis, see Louwerse et al., 2015). Other studies have shown that language statistics play an important role in facilitating access to perceptual knowledge when grounding is not afforded by the task context (Louwerse, 2018). The dimension of size is the focus of the current article.

Early proposals that physical size information is activated during lexical access were supported by demonstrations of *symbolic distance* effects. These studies reported that when participants were instructed to decide which of a pair of words denoted the larger or smaller object (e.g., *frog*/*wolf* vs. *frog*/*lobster*), response times were faster for pairs having a large difference in size (e.g., Moyer, 1973; Paivio, 1975 see Hoedemaker & Gordon, 2014). Later studies also reported size *congruity* effects. In these studies, words denoting large referents (e.g., *elephant*) printed in large font (congruent pairing) were responded to more quickly than when printed in small font (incongruent pairing), and vice versa (i.e., *ant* printed in small vs large font) when participants were instructed to judge either the size of the referent or the font (e.g., Rubinsten & Henik, 2002; Yao et al., 2022; but see Paivio, 1975). Size also influences performance on property verification tasks in which participants are presented with two words denoting an object referent and a feature and required to judge whether the latter is true of the former (e.g., HOUSE-*chimney*). These studies have shown that large properties are responded to more slowly (e.g., Solomon & Barsalou, 2004). Size knowledge has also been shown to influence memory performance. For example, the use of physical size judgements during the study/encoding phase (“Will this item fit into a shoebox?”; e.g., Healey & Kahana, 2014) has been shown to result in better retrieval of words denoting larger referents (e.g., Madan, 2021).

Mixed evidence that activation of physical size information during lexical access is not necessarily contingent on the goals of the task (i.e., the use of a size judgement) has come from studies employing lexical (word/nonword) decision tasks (LDTs). S. C. Sereno et al. (2009) were the first to report that responses were significantly faster for words with physically larger referents. However, neither Kang et al. (2011) nor Heard et al. (2019) were able to replicate the effect using megastudy data from the English Lexicon Project (ELP; Balota et al., 2007). Heard et al. observed a significant size effect for ELP accuracy (responses for words with larger referents were more accurate), while Kang et al. did not.

There is also evidence that “semantic size” (i.e., the size of the word’s referent) is a relevant dimension for both abstract and concrete words. Yao et al. (2013) found that abstract words whose referents were subjectively rated as being larger in size (e.g., *truth*) were responded to more quickly in the LDT than concepts rated as relatively small (e.g., *humble*). Using size ratings from their Glasgow norms and LDT latencies from the ELP, Scott et al. (2019) found an overall advantage for words denoting larger referents after controlling for concreteness and other lexical and semantic variables. Madan (2021) also recently showed an overall memory retrieval advantage for concrete words denoting larger referents that was not dependent on a concurrent size judgement task during encoding (i.e., participants were merely instructed to learn the words in a list; Lau et al., 2018). The advantage observed for “large” abstract words has been proposed to reflect a metaphorical association with the physical (especially visual) size of more concrete objects (Yao et al., 2022).

## Non-arbitrary relationships between size and sound in English words

The dimension of semantic size or magnitude is also represented in non-arbitrary sound-meaning associations across many spoken and signed languages (e.g., Blasi et al., 2016; Dingemanse et al., 2015; Nuckolls, 1999; Sidhu & Pexman, 2018; Winter & Perlman, 2021). These *iconic* (form resembles meaning) relationships include symbolic uses of vowel height/space, frequency/intonation (i.e., fundamental frequency, *f_o_*), word length and gesture space to denote the size of a word’s referent (Dingemanse et al., 2015). A century ago, Jespersen (1922; see also Newman, 1933) noted high vowels were more likely to be used in English words denoting smallness. Empirical investigations of “size sound symbolism” in spoken English began with Sapir (1929) who noted relationships between high vowels and small size (e.g., /i/ as in *teensy* and /ɪ/ as in *pill*) and low vowels and large size (e.g., /ɑ/ as in *tall*).

Sapir (1929) demonstrated that participants tended to associate nonwords, such as *mil* and *mal* with small and large objects, respectively. The “mil/mal effect” has been replicated multiple times (see Sidhu & Pexman, 2018; Winter & Perlman, 2021). Sapir proposed that the contraction of the vocal tract required to produce high vowels reflected a natural patterning of magnitude. Others have invoked relationships with acoustic frequencies of objects (small/large objects tend to resonate at high/low frequencies, respectively; see Spence, 2011) and animals (smaller animals typically produce higher pitch vocalisations; Hinton et al., 1994). The use of specific form features to represent size has also been referred to as *synaesthetic sound symbolism* and interpreted as evidence for grounding of word meaning in cross-modal simulations (e.g., Cuskley & Kirby, 2013; Hinton et al., 1994; Sidhu & Pexman, 2018).

However, there are some notable exceptions and limitations to size sound symbolism in English. For example, the adjectives *big* and *small* have high and low vowels, respectively, opposite to the predicted direction. Early attempts to confirm the mil/mal effect with real words were not particularly successful (e.g., Bentley & Varon, 1933; Brown, 1958 see Nuckolls, 1999, for a review). Sidhu and Pexman (2022) also recently revisited the mil/mal effect using real words and failed to observe a size congruency effect between vowel placement and object size. In addition, Winter and Perlman (2021) recently showed size symbolism was restricted to the phonemes /ɪ/, /i/, /ɑ/, and /t/ in adjectives in English, with no evidence for form-meaning associations in a larger set of words of various classes based on size ratings from the Glasgow norms (Scott et al., 2019).

Whereas sound symbolism tends to be limited to associations between sensory percepts and acoustic-phonetic properties of the speech signal, other non-arbitrary sound-meaning relationships, referred to as *form systematicity* or *typicality*, manifest more extensively within vocabularies as statistical regularities (Dingemanse et al., 2015). Multiple corpus and behavioural studies have demonstrated stress placement, syllable duration and vowel properties are probabilistic cues to syntactic category status (i.e., noun/verb) in English, being able to predict performance on various tasks, including grammatical category judgement, lexical decision, and reading aloud (e.g., Arciuli & Cupples, 2006; Cassidy & Kelly, 1991; de Zubicaray et al., 2021, 2023; Kelly, 1992; Monaghan et al., 2005, 2010; J. A. Sereno & Jongman, 1990; Sharpe & Marantz, 2017). Form typicality has also been demonstrated for concreteness (e.g., Reilly et al., 2017; Reilly & Kean, 2007; Westbury & Moroschan, 2009), a variable evaluating the degree to which a word is able to elicit a percept (Brysbaert et al., 2014). Dikker et al. (2010) observed significant modulation of the visual M100 response by syntactic form typicality during written word recognition in a magnetoencephalography study, leading them to conclude processing of form-meaning statistical regularities occurs rapidly and precedes semantic access.

## The present study

Despite the now extensive literature on size sound symbolism, to our knowledge, systematic mappings between surface form features and meaning for English size words are yet to be explored. We therefore conducted the present work to investigate whether statistical regularities in form-meaning mappings exist for English size words and, if they do, whether they can influence lexical access and memory. Specifically, we used a large set of surface form features to predict semantic size ratings of words in the Glasgow norms (Scott et al., 2019) and to calculate a measure of form typicality. Next, to determine whether form typicality can predict performance, we adopted the behavioural megastudy approach (Keuleers & Balota, 2015), using data sets for lexical decision and reading aloud from the ELP (Balota et al., 2007), auditory lexical decision from the Auditory English Lexicon Project (AELP; Goh et al., 2020), semantic (concrete/abstract) decision (Calgary Semantic Decision Project; Pexman et al., 2017), and recognition memory (Khanna & Cortese, 2021). For the latter investigations, we conducted a series of hierarchical regression analyses, entering a range of lexical and semantic control variables first, followed by size ratings, and finally form typicality.

## Study 1: investigating form-meaning mappings with size ratings of English words

**Figure 1.**
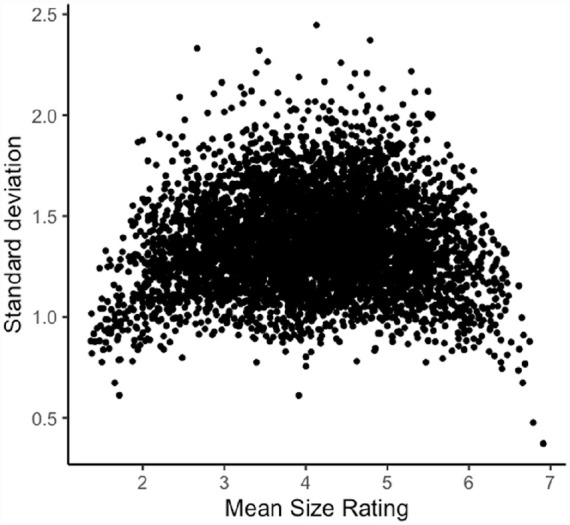
Size agreement in the Glasgow norms (*N* = 5,553).

In this study, our goal was to investigate systematic form-meaning associations for size using a large set of monosyllabic and multisyllabic words from the Glasgow norms (Scott et al., 2019). Participants rated semantic size on a 7-point scale ranging from *very small* to *very big*. However, Pollock (2018) elegantly demonstrated the problem of using average semantic ratings when participants’ judgements disagree, and this tends to occur particularly for words with mean ratings in the middle of a Likert-type scale. Figure 1 plots the mean size value and the standard deviation of every word rated in the Glasgow norms. It is evident from the plot that participants disagreed about the size dimension of many words (e.g., 27% of words have standard deviations exceeding 1.5). If there are systematic relationships between word forms and semantic size, then disagreements about (or substantial inter-individual variability in) ratings will likely obscure them (this is also the case for sound symbolism; cf., Winter & Perlman, 2021). Hence, for Study 1, we followed Pollock’s (2018) recommendation to select words with low rating standard deviations to ensure size judgements were in good agreement.

### Methods

#### Materials

The initial data set comprised the 5,553 English monosyllabic and multisyllabic words from the Glasgow size norms. Following Scott et al. (2019), we excluded items corresponding to the alternative meanings of homographs, then words with rating standard deviations greater than 1.5 (see Pollock, 2018), as well as proper names based on their Part of Speech (PoS) classification in UK English (van Heuven et al., 2014). For each word, we calculated 60 form variables, all of which were phonological/phonetic in nature excepting orthographic length, following Sharpe and Marantz’s (2017) approach. We used phonemic transcriptions from the Carnegie Mellon University (CMU) pronouncing dictionary along with stress category assignments from Tucker et al.’s (2019) database of 26,793 words. For each word, we coded whole word properties (length in letters, phonemes and syllables), initial and final phonemes, number of and initial and final positions for typical phonetic features (i.e., place and manner of articulation for consonants, place and height for vowels, voicing), and syllable position for primary stress: initial, final, medial (i.e., primary stress in the interior syllables), and multiple (i.e., more than one syllable with primary stress as per words with even stress). This resulted in a final set of 2,924 words common to all databases (959 monosyllabic, 1,236 disyllabic, 532 trisyllabic, 170 quadrisyllabic, 26 pentasyllabic, and 1 sexisyllabic).^1^

#### Design and analysis

All analyses were performed in R version 4.2.1 (R Core Team, 2022). The form variables were first evaluated for linear dependencies using the *caret* package (*findLinearCombos*; Kuhn, 2022), resulting in the exclusion of the following variables: *number low, number glottal, number glide, number voiceless, first phoneme is glide, first phoneme is voiceless, final phoneme is glottal, final phoneme is liquid, final phoneme is glide*, and *final phoneme is voiceless*. Next, to determine the best subset of form variables for predicting size ratings, we used the *leaps* package (Lumley, 2022). We selected the best fit model used Mallow’s *C_P_* criterion which is equivalent to Akaike’s information criterion (AIC; Boisbunon et al., 2014) to select the best fit model as it provides an estimate of a model’s *predictive accuracy* rather than its average likelihood (see Schmueli, 2010), consistent with our intention to provide a set of form typicality values for future studies to explore. Models with lower *C_P_* values are considered better in terms of both goodness of fit and complexity. Form typicality was calculated as the predicted value of the dependent variable for each word according to the model (i.e., form typicality = size + residual; e.g., Sharpe & Marantz, 2017). The values for all words were then Z-transformed, so that, positive values indicate larger size forms and negative values less so.

To determine whether our measure of form typicality differed according to the major lexical categories (adjectives, adverbs, function words, nouns, and verbs), we conducted an analysis of variance (ANOVA). Bartlett’s test showed the data violated the assumption of homogeneity of variance, χ^2^(4) = 23.521, *p* < .001. We therefore conducted a Welch’s ANOVA, followed by Games–Howell post hoc tests using the package *rstatix* (Kassambara, 2021). Violin plots were generated using the package *ggstatsplot* (Patil, 2021).

#### Transparency and openness

All data and analysis scripts for this and the subsequent regression studies are available for replication at: https://osf.io/k4yzf/.

### Results and discussion

The best fit model comprised 18 form variables, giving an adjusted *R*^2^ of .201 (Table 1).

**Table 1.** Best fit model for predicting size with form variables according to the *C_P_* criterion (*n* = 2,924).

Model comparison	Estimate	*SE*	*T*
(Intercept)	3.017	0.079	38.171***
Length	0.071	0.022	3.253**
Number high	–0.113	0.039	–2.936**
Number mid	–0.066	0.035	–1.917
Number labiodental	0.209	0.055	3.825***
Number linguodental	0.232	0.117	1.984*
Number alveolar	0.076	0.026	2.944**
Number palatal	0.230	0.040	5.792***
Number stop	–0.096	0.026	–3.643***
Number liquid	–0.066	0.033	–1.991*
First phoneme bilabial	–0.154	0.057	–2.721**
First phoneme labiodental	–0.199	0.090	–2.213*
First phoneme alveolar	–0.199	0.053	–3.774***
First phoneme palatal	–0.425	0.080	–5.340***
First phoneme voiced	0.126	0.044	2.855**
Final phoneme velar	0.126	0.070	1.800
Final phoneme stop	0.086	0.049	1.738
Number syllables	0.361	0.048	7.536***
Final stress position	0.239	0.051	4.721***

* *p* < .05. **p < .01. ***p < .001.

**Figure 2.**
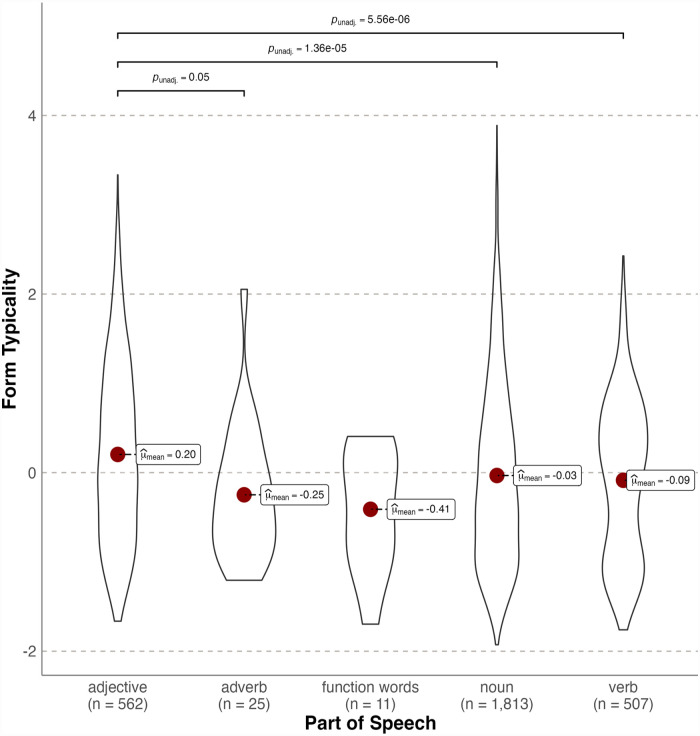
Violin plots showing probability densities of size form typicality standardised values as a function of lexical category. The red dot indicates the mean.

Figure 2 shows form typicality as a function of PoS (van Heuven et al., 2014). Typicality differed significantly according to lexical category, Welch’s *F*(4,57) = 8.843, *p* < .001, est. ω^2^ = 0.010). Post hoc Games–Howell tests revealed adjectives were significantly more typical forms than nouns (*M*_diff_ = 0.236, *p* < .001), verbs (*M*_diff_ = 0.289, *p* < .001), and adverbs (*M*_diff_ = 0.452, *p* < .05). All other comparisons were not significant (all *p*s > .05). Note that this pattern indicates form typicality for size differs to noun/verb (i.e., syntactic) form typicality in English (e.g., Sharpe & Marantz, 2017).

These results provide the first evidence of significant form typicality for English size words, showing systematic form-meaning mappings account for ~20% of variance in the Glasgow ratings. This contrasts with Winter and Perlman’s (2021) null findings for size sound symbolism in a comparably sized vocabulary using the same norms. However, adjectives (*N* = 562) were on average more typical forms than nouns (*N* = 1,813) and verbs (*N* = 507), consistent with Winter and Perlman’s (2021) finding of stronger size sound symbolism for adjectives.

## Study 2: written lexical decision

Study 2 investigated the extent to which size ratings and form typicality can predict written lexical decision performance from the ELP (Balota et al., 2007). S. C. Sereno et al. (2009, 2011) were the first to report that words with referents rated as large (e.g., *elephant*) were responded to more quickly in the LDT. A subsequent study by the same group (Yao et al., 2013) reported similar findings with abstract words. However, studies using the ELP megastudy data set and size ratings have produced less consistent results. For example, Kang et al. (2011) and Heard et al. (2019) were unable to replicate the effect for latencies, and Kang et al. did not observe a significant result for ELP accuracy, whereas Heard et al. did. All four studies used relatively small samples of words (90, 220, 324, and 618, respectively). Using a much larger sample of words from their Glasgow norms (*N* = 4,568), Scott et al. (2019) reported size was able to significantly predict ELP latencies. We therefore expected to replicate their findings using a similar hierarchical regression approach. *If* both size ratings and form typicality each contribute unique variance, we hypothesised that both size ratings and form typicality would be significant predictors of latencies and accuracy with the same set of words.

### Method

#### Materials

The initial data set comprised the 2,924 words from Study 1. We included the following lexical variables as predictors, orthographic length (New et al., 2006), orthographic Levenshtein distance (OLD), and phonological Levenshtein distance (PLD)—the mean number of steps required through letter and phoneme substitutions, insertions, or transpositions to transform a word into its 20 closest neighbours (OLD20 and PLD20; Suárez et al., 2011; Yarkoni et al., 2008), average bigram frequency (Gao et al., 2022), SUBTLEXus lexical frequencies expressed as a Zipf score (Brysbaert & New, 2009; Zipf, 1949), phonographic neighbourhood size—the number of words differing in one letter and one phoneme from a target word (Adelman & Brown, 2007), feedforward (spelling-to-sound), and feedback (sound-to-spelling) word onset token consistency (Chee et al., 2020), Age of acquisition (Scott et al., 2019), and prevalence—the number of people who know the word (Brysbaert et al., 2019). We also included three subjective semantic variables from the Glasgow norms following Yao et al.’s (2013) and Scott et al.’s (2019) analyses: concreteness, arousal (excitement vs calmness), and emotional valence (positive vs negative). All predictors were sourced from the South Carolina Psycholinguistic Database (SCOPE; Gao et al., 2022). Words in the ELP data set for which the above variables were available were included in the study (*N* = 2,829). Table 2 provides the descriptive statistics for each of the variables in the study.

**Table 2.** Descriptive statistics for the variables in Study 2 (*n* = 2,829).

Variable	*M*	*SD*
Length	6.36	2.01
OLD	2.23	0.76
PLD	2.11	0.88
Bigram frequency	1043.35	965.72
SUBTLEXus frequency	3.94	0.73
Phonographic neighbours	2.13	3.76
Feedforward consistency	0.87	0.17
Feedback consistency	0.84	0.18
Age of acquisition	4.10	1.19
Prevalence	2.32	0.20
Concreteness	4.41	1.39
Arousal	4.71	1.12
Valence	5.2	1.57
Size	4.18	1.06
Size form typicality	0.01	1.00
LDT latency (milliseconds)	664	76
LDT accuracy	0.96	0.06

OLD: orthographic Levenshtein distance; PLD: phonological Levenshtein distance; LDT: lexical (word/nonword) decision tasks.

#### Design and analysis

We examined the zero-order correlations between our size form typicality measure and the predictor variables and then performed separate hierarchical linear regressions with robust standard errors (Wilcox, 2016) with two dependent variables from the ELP: latencies standardised as *z*-scores (zRT) and mean accuracy; with the packages *estimatr* (Blair et al., 2022) and *lmtest* (Zeileis & Hothorn, 2002) in R version 4.2.1 (R Core Team, 2022). In each analysis, we entered the lexical and semantic predictor variables in Step 1, followed by size ratings in Step 2, then our measure of form typicality in Step 3. All predictor variables were mean-centred. Note that this method allows semantic size to explain both its unique and shared variance with form typicality. Hence, Step 3 provides a conservative estimate of the additional unique variance (if any) explained by form typicality.

### Results and discussion

**Figure 3.**
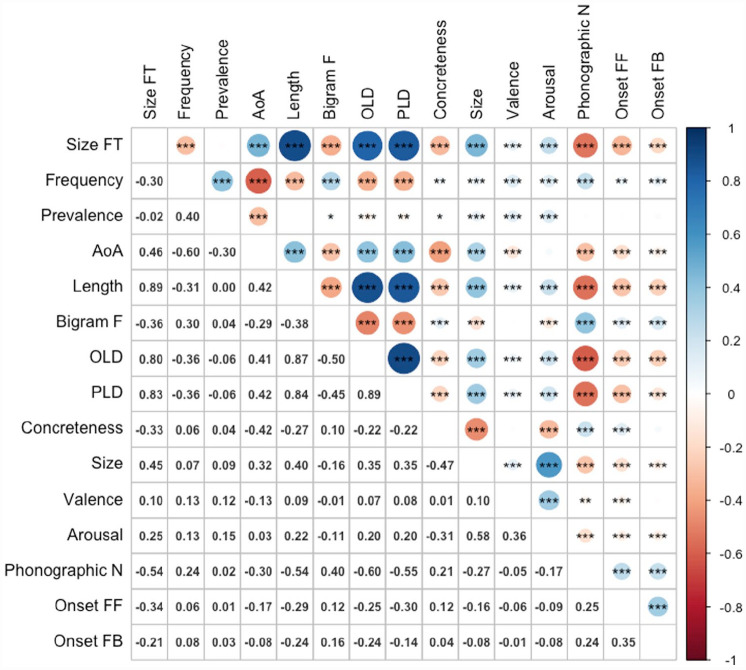
Correlations among variables (*n* = 2,829). Size FT: size form typicality; AoA: age of acquisition; bigram F: bigram frequency; OLD: orthographic Levenshtein distance; PLD: phonological Levenshtein distance; phonographic N: phonographic neighbourhood size; onset FF: feedforward (spelling-to-sound) onset consistency; onset FB: feedback (sound-to-spelling) onset consistency.

Figure 3 shows the zero-order Pearson correlations among the variables. Size form typicality showed significant positive correlations with length (*r* = .89, *p* < .001), age of acquisition (*r* = .46, *p* < .001), OLD (*r* = .80, *p* < .001), PLD (*r* = .83, *p* < .001), semantic size (*r* = .45, *p* < .001), valence (*r* = .10, *p* < .001), and arousal (*r* = .25, *p* < .001), and negative correlations with frequency (*r* = –.30, *p* < .001, bigram frequency (*r* = –.36, *p* < .001), phonographic neighbourhood size (*r* = –.54, *p* < .001), feedforward (spelling-to-sound) (*r* = –.34, *p* < .001) and feedback (sound-to-spelling) onset consistency (*r* = –.21, *p* < .001), and concreteness (*r* = –.33, *p* < .001). The only variable it did not correlate significantly with was word prevalence (*r* = –02). The results for latencies and accuracy for the ELP are presented in Tables 3 and 4, respectively.

**Table 3.** Regression coefficients from item-level analyses of English Lexicon Project LDT latencies (*n* = 2,829).

Model comparison	Estimate	*SE*	*t*	Adjusted *R*^2^	Δ*R*^2^
Step 1				.543***	
Length	0.024	0.003	9.110***		
OLD	0.027	0.009	2.877**		
PLD	0.020	0.007	2.935**		
Bigram frequency	8e–6	3e–6	2.899**		
SUBTLEXus Frequency	–0.071	0.005	–14.816***		
Phonographic neighbours	0.003	7e–4	3.933***		
Feedforward consistency	–0.017	0.015	–1.162		
Feedback consistency	0.001	0.015	0.080		
Age of acquisition	0.023	0.003	7.703***		
Prevalence	–0.204	0.016	–12.993***		
Concreteness	0.0008	0.002	0.377		
Arousal	–0.002	0.002	–0.783		
Valence	–0.003	0.002	–1.821		
Step 2				.543***	.00
Size	–0.005	0.003	–1.532		
Step 3				.544***	.001*
Size form typicality	0.012	0.006	1.970*		

LDT: lexical (word/nonword) decision task; OLD: orthographic Levenshtein distance; PLD: phonological Levenshtein distance.

* *p* < .05. **p < .01. ***p < .001.

**Table 4.** Regression coefficients from item-level analyses of English Lexicon Project LDT accuracy (*n* = 2,829).

Model comparison	Estimate	*SE*	*t*	Adjusted *R*^2^	Δ*R*^2^
Step 1				.363***	
Length	0.005	0.001	4.681***		
OLD	–0.013	0.003	–3.583***		
PLD	0.004	0.003	1.681		
Bigram frequency	–3e–6	1e–6	–2.820**		
SUBTLEXus frequency	0.016	0.002	9.420***		
Phonographic neighbours	–6e–4	3e–4	–1.676		
Feedforward consistency	0.005	0.006	0.832		
Feedback consistency	0.007	0.006	1.119		
Age of acquisition	–0.004	0.001	–3.451**		
Prevalence	0.145	0.012	12.584***		
Concreteness	–2e–4	8e–4	0.261		
Arousal	0.001	9e–4	1.224		
Valence	6e–5	6e–4	0.089		
Step 2				.363***	.000
Size	–5e–4	0.001	–0.365		
Step 3				.363***	.000
Size form typicality	.001	0.002	0.444		

LDT: lexical (word/nonword) decision task; OLD: orthographic Levenshtein distance; PLD: phonological Levenshtein distance.

* *p* < .05. **p < .01. ***p < .001.

Together, the lexical and semantic variables predicted ELP latencies and accuracy significantly, accounting for 54.3% and 36.3% of variance, respectively. Size ratings did not significantly predict either latencies or accuracy, replicating Kang et al.’s (2011) earlier null results with the ELP (see also Heard et al., 2019; cf., Scott et al., 2019). However, typicality significantly predicted an additional 0.1% of variance in latencies, with more typical forms associated with slower response times. Typicality did not significantly predict ELP accuracy.

## Study 3: auditory lexical decision

Our goal in Study 3 was to examine the extent to which size ratings and form typicality can predict auditory lexical decision performance from the AELP (Goh et al., 2020). Several studies have demonstrated that semantic variables, such as imageability, valence, and number of features are able to reliably predict auditory lexical decision performance (e.g., Gao et al., 2022; Goh et al., 2016; Sajin & Connine, 2014; Tyler et al., 2000; Wurm et al., 2004). However, to our knowledge, the question of whether size ratings can influence auditory lexical decision performance is yet to be investigated. Phonological typicality for syntactic category has been shown to be a significant predictor of auditory lexical decision performance (e.g., Sereno & Jongman, 1990; de Zubicaray et al., 2023). As above, we hypothesised that both size ratings and form typicality would be significant predictors of latencies and accuracy in auditory lexical decision *if* they each contributed unique variance. We expected form typicality would predict more variance in performance than in Study 2 given the use of auditory stimuli.

### Method

#### Materials

The initial data set comprised the 2,924 words from Study 1. We included identical variables as control predictors to Study 2, with the addition of American dialect auditory token duration in milliseconds (Goh et al., 2020). All predictors were again sourced from SCOPE (Gao et al., 2022). Words in the AELP data set for which the above variables were available were included in the study (*N* = 2,266). Table 5 provides the descriptive statistics for each of the variables in the study.

**Table 5.** Descriptive statistics for the variables in Study 3 (*n* = 2,226).

Variable	*M*	*SD*
Length	6.10	1.90
Duration	776	128
OLD	2.20	0.73
PLD	2.00	0.85
Bigram frequency	1059	985
SUBTLEXus frequency	4.00	0.70
Phonographic neighbours	2.30	4.00
Feedforward consistency	0.87	0.17
Feedback consistency	0.84	0.18
Age of acquisition	4.00	1.20
Prevalence	2.30	0.17
Concreteness	4.50	1.40
Arousal	4.70	1.10
Valence	5.20	1.50
Size	4.10	1.10
Size form typicality	-0.07	0.99
LDT latency (milliseconds)	946	86
LDT accuracy	0.95	0.07

OLD: orthographic Levenshtein distance; PLD: phonological Levenshtein distance; LDT: lexical (word/nonword) decision task.

#### Design and analysis

Identical to Study 2, except that the two dependent variables from the AELP were American dialect latencies standardised as *z*-scores (zRT) and mean accuracy.

### Results and discussion

**Figure 4.**
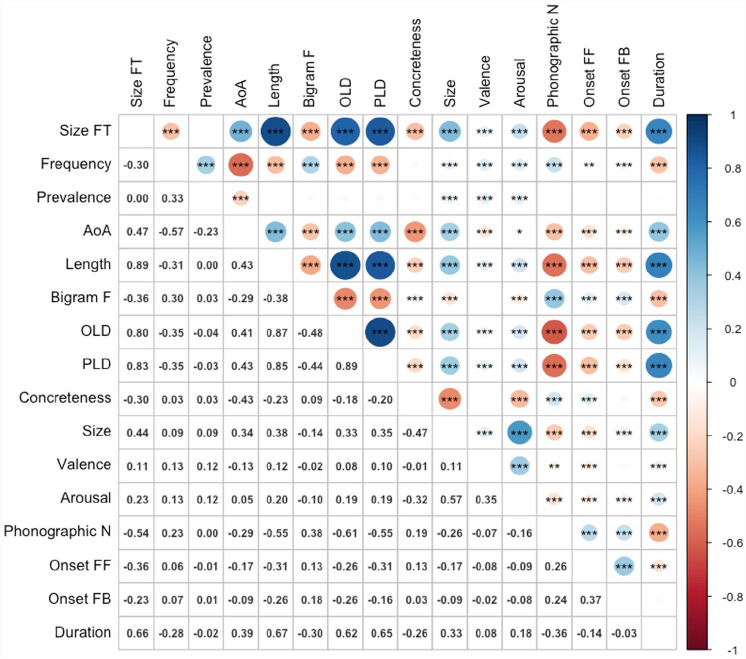
Correlations among variables (*n* = 2,266). Size FT: size form typicality; AoA: age of acquisition; bigram F: bigram frequency; OLD: orthographic Levenshtein distance; PLD: phonological Levenshtein distance; phonographic N: phonographic neighbourhood size; onset FF: feedforward (spelling-to-sound) onset consistency; onset FB: feedback (sound-to-spelling) onset consistency.

Figure 4 shows the zero-order Pearson correlations among the variables. The magnitudes and directions of relationships between size form typicality and the predictor variables were virtually identical to Study 2, with the addition of a positive correlation with token duration (*r* = .66, *p* < .001). The results for latencies and accuracy are presented in Tables 6 and 7, respectively.

**Table 6.** Regression coefficients from item-level analyses of Auditory English Lexical Decision Project latencies (*n* = 2,266).

Model comparison	Estimate	*SE*	*t*	Adjusted *R*^2^	Δ*R*^2^
Step 1				.474***	
Length	–0.008	0.006	–1.461		
Duration	0.002	0.000	33.761***		
OLD	0.033	0.019	1.668		
PLD	–0.100	0.015	–6.658***		
Bigram frequency	0.000	0.000	1.032		
SUBTLEXus frequency	–0.044	0.010	–4.440***		
Phonographic neighbours	0.009	0.002	5.356***		
Feedforward consistency	0.008	0.040	0.189		
Feedback consistency	0.016	0.036	0.446		
Age of acquisition	0.035	0.006	5.594***		
Prevalence	–0.195	0.035	–5.533***		
Concreteness	0.018	0.004	4.117***		
Arousal	–0.034	0.005	–6.273***		
Valence	0.005	0.003	1.372		
Step 2				.476***	.002*
Size	0.017	0.007	2.461*		
Step 3				.482***	.006***
Size form typicality	–0.067	0.012	–5.592***		

OLD: orthographic Levenshtein distance; PLD: phonological Levenshtein distance.

* *p* < .05. **p < .01. ***p < .001.

**Table 7.** Regression coefficients from item-level analyses of Auditory English Lexical Decision Project accuracy (*n* = 2,266).

Model comparison	Estimate	*SE*	*t*	Adjusted *R*^2^	Δ*R*^2^
Step 1				.191***	
Length	0.008	0.002	5.245***		
Duration	0.000	0.000	0.848		
OLD	–0.019	0.005	–3.911***		
PLD	0.022	0.004	5.536***		
Bigram frequency	0.000	0.000	–1.577		
SUBTLEXus frequency	0.015	0.003	5.517***		
Phonographic neighbours	0.000	0.000	–0.480		
Feedforward consistency	0.006	0.011	0.540		
Feedback consistency	–0.009	0.009	–0.995		
Age of acquisition	–0.009	0.002	–4.775		
Prevalence	0.086	0.015	5.609***		
Concreteness					
Arousal	0.006	0.002	3.729***		
Valence	–0.002	0.001	–2.384*		
Step 2				.192***	.001
Size	–0.003	0.002	–1.684		
Step 3				.194***	.002**
Size form typicality	0.010	0.003	3.034**		

OLD: orthographic Levenshtein distance; PLD: phonological Levenshtein distance.

* *p* < .05. **p < .01. ***p < .001.

Combined, the lexical and semantic variables predicted AELP latencies and accuracy significantly, accounting for 47.4% and 19.1% of variance, respectively. Size was a significant predictor of latencies, explaining an additional 0.2% of variance, but not of accuracy. However, form typicality significantly predicted 0.6% and 0.2% of variance in AELP latencies and accuracy, respectively. More typical forms were responded to more quickly and accurately.

## Study 4: reading aloud

Study 4 explored the extent to which size ratings and form typicality can predict reading aloud performance (i.e., word naming/pronunciation) from the ELP (Balota et al., 2007). To our knowledge, whether size ratings can influence reading aloud performance is yet to be investigated. As per our previous studies, we hypothesised that both size ratings and form typicality would be significant predictors of latencies and accuracy with the same set of words *if* they each contributed unique variance.

### Method

#### Materials

The data set comprised the same set of 2,829 words from Study 2. Predictor variables were identical to Study 2.

#### Design and analysis

Identical to Study 2, except that the two dependent variables from the ELP were for reading aloud: latencies standardised as *z*-scores (zRT) and mean accuracy.

### Results and discussion

The results for latencies and accuracy are presented in Tables 8 and 9, respectively.

**Table 8.** Regression coefficients from item-level analyses of English Lexicon Project reading aloud latencies (*n* = 2,829).

Model comparison	Estimate	*SE*	*t*	Adjusted *R*^2^	Δ*R*^2^
Step 1				.430***	
Length	0.029	0.053	21.166***		
OLD	–0.011	0.009	–1.235		
PLD	0.012	0.007	1.708		
Bigram frequency	–2e–6	3e–6	0.852		
SUBTLEXus frequency	–0.043	0.004	–9.651***		
Phonographic neighbours	–8e–4	7e–4	–1.134		
Feedforward consistency	–0.109	0.016	–6.626***		
Feedback consistency	0.006	0.015	0.382		
Age of acquisition	0.017	0.003	5.652***		
Prevalence	–0.156	0.017	–9.203***		
Concreteness	0.002	0.002	0.930		
Arousal	–0.001	0.003	0.523		
Valence	–0.001	0.002	–1.341		
Step 2				.431***	.001*
Size	–0.008	0.003	–2.260*		
Step 3				.436***	.005***
Size form typicality	–0.028	0.006	–4.574***		

OLD: orthographic Levenshtein distance; PLD: phonological Levenshtein distance;

* *p* < .05. **p < .01. ***p < .001.

**Table 9.** Regression coefficients from item-level analyses of English Lexicon Project reading aloud accuracy (*n* = 2,829).

Model comparison	Estimate	*SE*	*t*	Adjusted *R*^2^	Δ*R*^2^
Step 1				.158***	
Length	–6e–5	6e–4	–0.089		
OLD	0.005	0.002	1.883		
PLD	–0.006	0.002	–3.153**		
Bigram frequency	–2e–6	5e–7	–3.547***		
SUBTLEXus frequency	0.003	0.001	2.727**		
Phonographic neighbours	4e–4	2e–4	2.248*		
Feedforward consistency	0.006	0.004	1.645		
Feedback consistency	0.005	0.003	1.333		
Age of acquisition	–0.003	7e–4	–5.150***		
Prevalence	0.047	0.008	5.940***		
Concreteness	–3e–4	5e–4	0.764		
Arousal	8e–4	6e–4	1.282		
Valence	–4e–4	4e–4	–0.836		
Step 2				.158***	.000
Size	–9e–4	8e–4	–1.104		
Step 3				.158***	.000
Size form typicality	–3e–4	0.001	–0.253		

OLD: orthographic Levenshtein distance; PLD: phonological Levenshtein distance;

* *p* < .05. **p < .01. ***p < .001.

Together, the lexical and semantic variables predicted ELP reading aloud latencies and accuracy significantly, accounting for 43% and 15.8% of variance, respectively. Size ratings and form typicality significantly predicted 0.1% and 0.5% of variance in latencies, respectively. Words with larger referents and more typical forms were produced more quickly. However, neither significantly predicted accuracy.

## Study 5: semantic decision

Recently, Yao et al. (2022; Experiment 3) reported size congruity effects for both concrete and abstract words, a finding they interpret as compatible with the grounded/embodied cognition framework (e.g., Barsalou, 2008). In Study 5, we therefore investigated the extent to which semantic size and its corresponding form typicality measure could predict semantic (concrete/abstract) decision latency and accuracy from the Calgary Semantic Decision Project (CSDP; Pexman et al., 2017). To our knowledge, no study has yet explored whether semantic size influences CSDP performance, although Lupyan and Winter (2018) recently reported that higher iconicity resulted in *poorer* accuracy for abstract words using the CSDP data set. We hypothesised semantic size would predict more variance than form typicality in semantic category decision performance given the explicit requirements of the task to direct attention to conceptual features.

### Method

#### Materials

The initial data set comprised the same set of 2,924 words from Study 1. Predictor variables were identical to Studies 2–4. Words in the CSDP data set for which the predictor variables were available were included in the study (*N* = 1,006). Table 10 provides the descriptive statistics for each of the variables in the study.

**Table 10.** Descriptive statistics for the variables in Study 5 (*n* = 1,006).

Variable	*M*	*SD*
Length	7.00	1.80
OLD	2.50	0.75
PLD	2.40	0.88
Bigram frequency	839	843
SUBTLEXus frequency	3.70	0.71
Phonographic neighbours	1.00	2.40
Feedforward consistency	0.85	0.17
Feedback consistency	0.82	0.18
Age of acquisition	4.40	1.20
Prevalence	2.30	0.22
Concreteness	4.20	1.50
Arousal	4.80	1.10
Valence	5.30	1.60
Size	4.30	1.00
Size form typicality	0.34	0.90
SDT latency (milliseconds)	949	162
SDT accuracy	0.88	0.15

OLD: orthographic Levenshtein distance; PLD: phonological Levenshtein distance; SDT: semantic decision task.

#### Design and analysis

Identical to Studies 2–4, with the exception that we analysed responses for concrete and abstract words separately given the evidence that participants adopt different criteria for them (Pexman & Yap, 2018; Pexman et al., 2018) and Lupyan and Winter’s (2018) finding that iconicity influences abstract word decisions. The two dependent variables were latencies standardised as *z*-scores (zRT) and mean accuracy from the CSDP semantic decision task.

### Results and discussion

**Figure 5.**
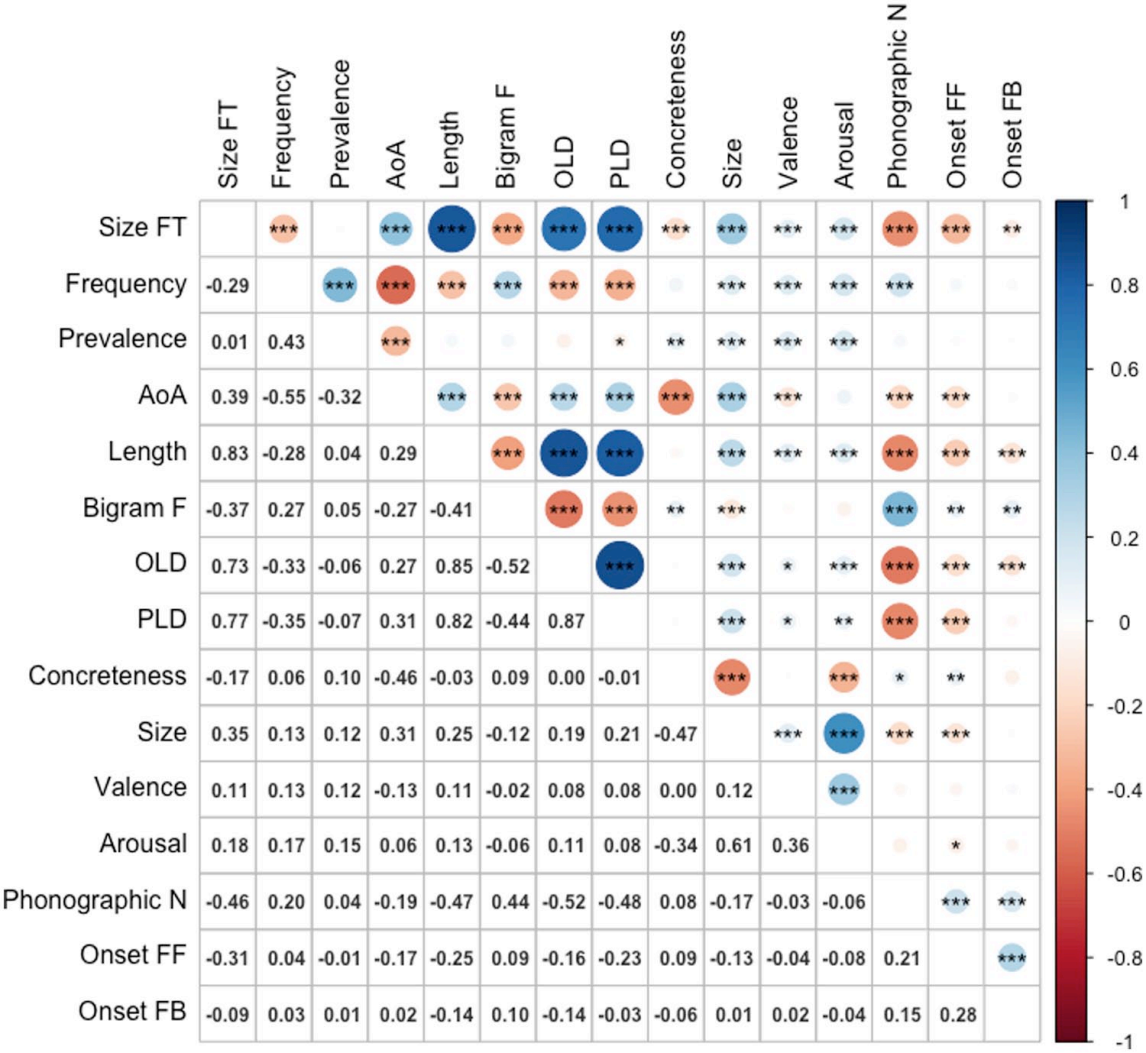
Correlations among variables (*n* = 1,006). Size FT: size form typicality; AoA: age of acquisition; bigram F: bigram frequency; OLD: orthographic Levenshtein distance; PLD: phonological Levenshtein distance; phonographic N: phonographic neighbourhood size; onset FF: feedforward (spelling-to-sound) onset consistency; onset FB: feedback (sound-to-spelling) onset consistency.

Figure 5 shows the zero-order Pearson correlations among the variables. The magnitudes and directions of relationships between size form typicality and the predictor variables were similar to the previous studies. The results for latencies and accuracy for concrete and abstract words are presented in Tables 11 and 12, and 13 and 14, respectively.

**Table 11.** Regression coefficients from item-level analyses of Calgary Semantic Decision Project latencies for concrete words (*n* = 475).

Model comparison	Estimate	*SE*	*t*	Adjusted *R*^2^	Δ*R*^2^
Step 1				.534***	
Length	0.088	0.019	4.530***		
OLD	–0.095	0.046	–2.068*		
PLD	–0.012	0.036	–0.342		
Bigram frequency	0.000	0.000	–0.009		
SUBTLEXus Frequency	–0.089	0.024	–3.767***		
Phonographic neighbours	0.009	0.005	1.637		
Feedforward consistency	–0.065	0.096	–0.677		
Feedback consistency	0.194	0.071	2.742**		
Age of acquisition	0.049	0.017	2.942**		
Prevalence	–0.161	0.086	–1.862		
Concreteness	–0.278	0.022	–12.497***		
Arousal	–0.005	0.015	–0.337		
Valence	–0.037	0.013	–2.864**		
Step 2				.536***	.002*
Size	0.029	0.015	1.962^†^		
Step 3					
Size form typicality				.537***	.001
	0.051	0.033	1.550		

OLD: orthographic Levenshtein distance; PLD: phonological Levenshtein distance.

† *p* = .05. *p < .05. **p < .01. ***p < .001.

**Table 12.** Regression coefficients from item-level analyses of Calgary Semantic Decision Project accuracy for concrete words (*n* = 475).

Model comparison	Estimate	*SE*	*t*	Adjusted *R*^2^	Δ*R*^2^
Step 1				.414***	
Length	–0.010	0.007	–1.506		
OLD	0.009	0.016	0.590		
PLD	–0.003	0.012	–0.292		
Bigram frequency	0.000	0.000	–0.063		
SUBTLEXus frequency	–0.009	0.011	–0.811		
Phonographic neighbours	–0.007	0.004	–1.717		
Feedforward consistency	0.032	0.036	0.897		
Feedback consistency	–0.025	0.024	–1.043		
Age of acquisition	0.002	0.006	0.345		
Prevalence	0.055	0.033	1.646		
Concreteness	0.113	0.010	10.813***		
Arousal	0.008	0.006	1.283		
Valence	0.008	0.005	1.586		
Step 2				.414***	.000
Size	–0.005	0.005	–0.910		
Step 3				.420	.006**
Size form typicality	–0.030	0.011	–2.776**		

OLD: orthographic Levenshtein distance; PLD: phonological Levenshtein distance.

*p < .05. **p < .01. ***p < .001.

**Table 13.** Regression coefficients from item-level analyses of Calgary Semantic Decision Project latencies for abstract words (*n* = 531).

Model comparison	Estimate	*SE*	*t*	Adjusted *R*^2^	Δ*R*^2^
Step 1				.187***	
Length	0.008	0.013	0.604		
OLD	–0.026	0.040	–0.653		
PLD	0.036	0.028	1.255		
Bigram frequency	0.000	0.000	0.785		
SUBTLEXus frequency	–0.063	0.025	–2.528*		
Phonographic neighbours	0.009	0.005	1.670		
Feedforward consistency	–0.008	0.081	–0.095		
Feedback consistency	–0.086	0.082	–1.044		
Age of acquisition	0.031	0.018	1.681		
Prevalence	–0.129	0.061	–2.133*		
Concreteness	0.202	0.025	8.068***		
Arousal	–0.006	0.011	–0.581		
Valence	–0.001	0.006	–0.230		
Step 2				.190***	.003
Size	0.036	0.023	1.576		
Step 3				.213***	.023***
Size form typicality	–0.101	0.025	–4.020***		

OLD: orthographic Levenshtein distance; PLD: phonological Levenshtein distance;

* *p* < .05. **p < .01. ***p < .001.

**Table 14.** Regression coefficients from item-level analyses of Calgary Semantic Decision Project accuracies for abstract words (*n* = 531).

Model comparison	Estimate	*SE*	*t*	Adjusted *R*^2^	Δ*R*^2^
Step 1				.123***	
Length	0.001	0.008	0.145		
OLD	–0.002	0.020	–0.103		
PLD	0.000	0.000	–0.003		
Bigram frequency	0.000	0.000	–0.512		
SUBTLEXus frequency	–0.039	0.019	–2.061*		
Phonographic neighbours	–0.013	0.004	–3.505***		
Feedforward consistency	0.005	0.046	0.103		
Feedback consistency	0.067	0.065	1.025		
Age of acquisition	–0.004	0.008	–0.512		
Prevalence	0.035	0.030	1.152		
Concreteness	–0.083	0.016	–5.187***		
Arousal	0.001	0.006	0.194		
Valence	0.002	0.004	0.419		
Step 2				.123***	.000
Size	–0.013	0.013	–1.040		
Step 3				.127***	.004
Size form typicality	0.024	0.013	1.828		

OLD: orthographic Levenshtein distance; PLD: phonological Levenshtein distance;

* *p* < .05. **p < .01. ***p < .001.

Together, the lexical and semantic variables predicted CSDP latencies and accuracy for concrete words significantly, accounting for 53.4% and 41.2% of variance, respectively. Size approached significance as a predictor of latencies (*p* = .05, 0.2% of variance) with words denoting larger constructs being responded to more quickly, although size did not significantly predict accuracy. Size form typicality was not a significant predictor of latencies but did significantly predict accuracy, explaining 0.6% of variance, with more typical forms being responded to less accurately.

The pattern of results for abstracts words differed to that for concrete words. Together, the lexical and semantic variables predicted CSDP latencies and accuracy significantly, accounting for approximately 18.7% of variance in the former and 12.3% in the latter. Surprisingly, size was not a significant predictor of either latencies or accuracy. Size form typicality was a significant predictor of latencies, accounting for an additional 2.3% of variance. More typical forms were associated with faster abstract responses. However, form typicality did not significantly predict accuracy.

## Study 6: recognition memory

Many studies of recall and recognition have demonstrated that various word properties, including lexical frequency, age of acquisition, concreteness, and arousal are able to influence their memorability (see Khanna & Cortese, 2021; Lau et al., 2018; Madan, 2021). The use of physical size judgements during encoding has been shown to result in better memory for words denoting larger referents (e.g., Madan, 2021). Madan (2021) also demonstrated a memory advantage using size ratings without any judgements employed at encoding (i.e., participants were merely instructed to remember the words).

Study 6 therefore explored the extent to which size ratings and form typicality can predict recognition memory performance using Khanna and Cortese’s (2021) megastudy data set combining monosyllabic (Cortese et al., 2010) and disyllabic (Cortese et al., 2015) word lists. In both studies, participants were required to learn lists of words, with each word presented individually for a brief period without a concurrent encoding task. As per our previous studies, we hypothesised that both size ratings and form typicality would be significant predictors of performance with the same set of words *if* they each contributed unique variance.

### Method

#### Materials

The initial data set comprised the same set of 2,924 words from Study 1. Predictor variables were identical to Studies 2 and 4. Monosyllabic (*N* = 822) and disyllabic (*N* = 833) words for which these variables were available in Khanna and Cortese’s (2021) database were included in the study. Table 15 provides the descriptive statistics for each of the variables in the study.

**Table 15.** Descriptive statistics for the variables in Study 6 (*n* = 1,655).

Variable	*M*	*SD*
Length	5.32	1.23
OLD	1.87	0.50
PLD	1.68	0.54
Bigram frequency	1,212.65	1,069.18
SUBTLEXus frequency	4.11	0.69
Phonographic neighbours	3.09	4.39
Feedforward consistency	0.90	0.17
Feedback consistency	0.86	0.20
Age of acquisition	3.83	1.14
Prevalence	2.33	0.18
Concreteness	4.64	1.36
Arousal	4.57	1.06
Valence	5.14	1.41
Size	3.94	1.04
Size form typicality	–0.49	0.68
Corrected hit probability	0.5	0.13

OLD: orthographic Levenshtein distance; PLD: phonological Levenshtein distance.

#### Design and analysis

Identical to Study 2, except that the dependent variable was corrected hit probability (hits minus false alarms).

### Results and discussion

**Figure 6.**
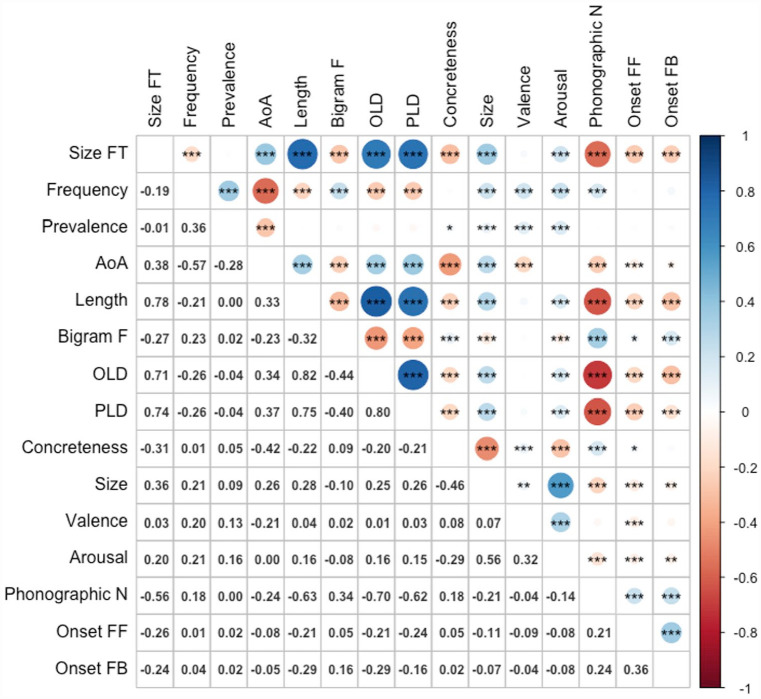
Correlations among variables (*n* = 1,006). Size FT: size form typicality; AoA: age of acquisition; Bigram F: bigram frequency; OLD: orthographic Levenshtein distance; PLD: phonological Levenshtein distance; phonographic N: phonographic neighbourhood size; onset FF: feedforward (spelling-to-sound) onset consistency; onset FB: feedback (sound-to-spelling) onset consistency.

Figure 6 shows the zero-order Pearson correlations among the variables. The magnitudes and directions of relationships between size form typicality and the predictor variables were similar to the previous studies, although valence no longer showed a significant correlation with form typicality for size. The results for corrected hit probability are shown in Table 16. The lexical and semantic variables together significantly predicted recognition memory performance, accounting for 30% of the variance. Size ratings and form typicality both significantly predicted performance, accounting for 0.4% and 0.1% of variance, respectively. Words denoting larger referents and forms more typical of larger referents resulted in better memory.

**Table 16.** Regression coefficients from item-level analyses of recognition memory (*n* = 1,655).

Model comparison	Estimate	*SE*	*t*	Adjusted *R*^2^	Δ*R*^2^
Step 1				.300***	
Length	–0.036	0.004	–9.174***		
OLD	0.096	0.011	8.439***		
PLD	–0.003	0.009	–0.312		
Bigram frequency	9e–6	3e–6	3.499***		
SUBTLEXus frequency	–0.048	0.005	–9.002***		
Phonographic neighbours	6e-4	8e-4	0.732		
Feedforward consistency	–0.011	0.017	–0.648		
Feedback consistency	–0.045	0.016	–2.813**		
Age of acquisition	–0.002	0.004	–0.425		
Prevalence	–0.046	0.017	–2.614**		
Concreteness	0.043	0.003	17.218***		
Arousal	0.022	0.003	7.313***		
Valence	–0.005	0.002	–2.085*		
Step 2				.304***	.004***
Size	–0.013	0.003	–3.594***		
Step 3				.305***	.001*
Size form typicality	–0.014	0.007	–1.996*		

OLD: orthographic Levenshtein distance; PLD: phonological Levenshtein distance;

* *p* < .05. **p < .01. ***p < .001.

## General discussion

In the present article, we investigated non-arbitrary statistical relationships between the surface form properties of English words and ratings of their semantic size. Our results provide the first evidence for significant form typicality for size words. In five investigations of behavioural megastudy data sets, we found that form typicality was a significant predictor of lexical access during word comprehension and production as well as recognition memory. Furthermore, form typicality was a stronger and more consistent predictor of performance across experiments than semantic size. Although the effects of form typicality we observed in the megastudy data sets might seem relatively modest, it is worth noting that they were as large or larger than the effects reported for most semantic variables in written and spoken word recognition, many of which have been interpreted as evidence for grounded cognition (e.g., Goh et al., 2016; Juhasz & Yap, 2013; Pexman et al., 2008). Furthermore, they are likely to be conservative estimates given we entered form typicality after all other variables in our regression models.

Study 1 demonstrated that surface form variables are significant predictors of English words’ rated size dimensions, explaining approximately 20% of the variance in the Glasgow norms (Scott et al., 2019). Given that Winter and Perlman (2021) failed to find any evidence for size sound symbolism in a comparable sample of words of various classes using the same Glasgow norms, our positive finding is consistent with the proposal that form typicality and iconicity are distinct constructs (e.g., Dingemanse et al., 2015). This likely reflects the fact that size sound symbolism is largely concerned with vowels, while statistical regularities encompass phonetic features, syllable stress patterns, and phonotactic constraints (see Sharpe & Marantz, 2017). Study 1 revealed that words rated as being larger in size tend to be longer and have more syllables, with lexical stress more likely to be applied in the final position. They also comprise fewer higher and mid placed vowel sounds. They had more dental, alveolar and palatal sounds, and fewer stops and liquids. Initial phonemes contributed via voicing and comprised fewer bilabial, labiodental, alveolar, or palatal sounds. Final phonemes tended to have more stop and velar sounds.

We also found that form typicality varied according to part-of-speech, with adjectives being more typical forms than other classes, similar to Winter and Perlman’s (2021) findings for size sound symbolism. Although adjectives were on average more typical forms, the majority of the words in our sample from the Glasgow norms were nouns (*N* = 1,813), followed by adjectives (*N* = 562) and verbs (*N* = 507). To investigate whether our measure of size form typicality was influenced by typicality for noun/verb status (e.g., Arciuli & Cupples, 2006; Arciuli & Monaghan, 2009; Kelly, 1992; Monaghan et al., 2005; Sharpe & Marantz, 2017), we calculated the correlation between Sharpe and Marantz’s (2017) measure of form typicality for syntactic category and our measure of size typicality in a subset of monosyllabic and disyllabic nouns and verbs in both studies (*N* = 1,271). This revealed a negligible and non-significant positive correlation, *r* = 0.042 (*p* = .131). Hence, size form typicality is clearly distinct from phonological typicality for noun/verb status.

We were unable to replicate recent reports of size ratings predicting ELP performance (e.g., Heard et al., 2019; Scott et al., 2019) although our null results for latencies and accuracy were consistent with an earlier report by Kang et al. (2011; see also Heard et al., 2019 for a null result for latencies). Possible explanations for these differing results include our use of only words with good rating agreement (Pollock, 2018), and our selection of lexical control variables (see Yao et al., 2022). Whereas, Scott et al. (2019) and Heard et al. (2019) included length, frequency, and age of acquisition as lexical control variables, we additionally included well-established measures of orthographic and phonological neighbourhood distances and spelling-sound consistency, consistent with Kang et al.’s (2011) approach, in addition to word prevalence (Brysbaert et al., 2019). By contrast, form typicality was a significant predictor of ELP latencies, with more typical forms for larger sizes slowing responses. This was despite form typicality being entered after size ratings in the regression analyses. However, the effect was relatively weak (0.1% of variance explained).

The results from Study 3 for auditory lexical decision from the AELP showed size ratings significantly predicted latencies, explaining a small proportion of variance (0.1%). Form typicality again predicted a significant proportion of variance in latencies (0.8%) as well as accuracy (0.3%). Unlike Study 2, forms typical of larger size were associated with faster and more accurate responses. Both size ratings and form typicality emerged as significant predictors of reading aloud latencies in Study 4, with the latter accounting for more variance. In addition, both variables facilitated responses, although the proportion of variance accounted for was again quite small (0.1% and 0.5%). The findings from Studies 2–4 indicate that the influence of form typicality outweighs that of semantic size during lexical access in word recognition and production.

Our sole finding of a borderline significant contribution of semantic size to predicting latencies for concrete category decisions (0.2%) in Study 5 is surprising given proposals that size information is activated according to the conceptual demands of the task (cf., Yao et al., 2022). However, size form typicality was able to significantly predict 0.6% of variance in accuracy for concrete words and 2.3% of variance in latencies for abstract words. When participants encountered form information in concrete words that was more typical of large-sized referents, they made more incorrect “abstract” decisions. In addition, when they encountered this information in abstract words, they made faster “abstract” decisions. Thus, knowledge about form typicality for size informs semantic category decisions and seems particularly relevant for processing abstract concepts. Interestingly, this pattern differs to that reported by Lupyan and Winter (2018), as more iconic abstract words were more likely to be mistaken for concrete words in their study, again suggesting that form typicality and iconicity are distinct constructs (e.g., Dingemanse et al., 2015). The non-significant to weak contributions of semantic size across our Studies 2–5 support Hoedemaker and Gordon’s (2014) conclusion that activation/simulation of semantic size is more goal-driven than automatic during lexical access, challenging strong accounts of grounded cognition. There is more robust evidence that semantic size influences performance on tasks involving judgements that explicitly require participants to access size knowledge (e.g., Hoedemaker & Gordon, 2014; Paivio, 1975; Rubinsten & Henik, 2002; Solomon & Barsalou, 2004; Yao et al., 2022).

Recognition memory was significantly predicted by both semantic size and form typicality in Study 6. However, words denoting larger referents and their more typical forms were *less* likely to be remembered. The direction of the effect we observed for size ratings in memory retrieval was opposite to that reported recently by Madan (2021), the reason for which is not entirely clear. While Madan (2021) examined the effects of various lexical and semantic variables on item recall rather than recognition, most models of memory assume common mechanisms underlie both tasks (e.g., de Zubicaray et al., 2007; Kahana, 2012; Kahana et al., 2005). The directions of the effects we observed for other predictor variables were consistent with those reported reliably in the literature, such as low-frequency, more concrete, and arousing words resulting in better recognition (e.g., de Zubicaray et al., 2005; Glanzer & Adams, 1985; Khanna & Cortese, 2021; Mather, 2007). A possible explanation for the discrepancy is that Madan (2021) used Lau et al.’s (2018) megastudy data set that was restricted to a relatively small set of concrete nouns (*N* = 532), whereas, we used a much larger sample of both abstract and concrete words of various classes (*N* = 1,655) from Khanna and Cortese’s (2021) megastudy. To test this hypothesis, we repeated the analysis restricting the data set to only nouns with Glasgow concreteness ratings of five or greater (*N* = 634). The direction of effect remained the same (i.e., nouns denoting larger referents were still *less* likely to be remembered). Another possible explanation for the discrepancy between studies is that Madan’s (2021) measure of size was based on a probability distribution of responses for a binary judgement (“Will this item fit into a shoebox?”; e.g., Healey & Kahana, 2014) rather than on a Likert-type scale per the Glasgow norms (Scott et al., 2019). Nonetheless, size ratings and form typicality contributed only a small proportion of variance (0.5% overall).

When viewed together, the results from the megastudy data sets show that the effect of form typicality for size is facilitatory on tasks requiring parsing/assembly of phonological/phonetic information in perception and production (i.e., auditory lexical decision and reading aloud), but inhibitory on tasks requiring orthographic processing (lexical decision and recognition memory of written words) in which accessing phonology is not obligatory. These findings for form typicality resemble those reported for phonological neighbourhood density for which facilitatory effects are reported more frequently for production and inhibitory effects reported more often in visual lexical decision (e.g., Grainger et al., 2005; Vitevitch & Luce, 2016). Hence, the effects of form typicality we observed might be akin to neighbourhood density effects. Inhibitory effects observed for visual word processing might also reflect the additional time needed to engage grapheme-to-phoneme conversion mechanisms when potentially meaningful form information is recognised from a word’s spelling. Note that as we included measures of phonographic neighbourhood size, Levenshtein distances for orthographic and phonological neighbourhoods, and feedforward and feedback spelling-to-sound consistency as control predictors, the form typicality effects we observed cannot be attributed to these variables. Nor can these effects be attributed to grounding or simulation of size knowledge in sensorimotor systems, as has been proposed for size sound symbolism (e.g., Cuskley & Kirby, 2013; Hinton et al., 1994; Sidhu & Pexman, 2018). Rather, they are more likely to reflect statistical knowledge acquired about the English language (e.g., Arciuli, 2017). Here, size form typicality was significantly associated with words learned *later* in life, unlike iconic relationships that are typically learned earlier (e.g., Perry et al., 2015).

How might non-arbitrary form-meaning associations influence performance on language processing tasks? Lupyan and Winter (2018) proposed that more iconic words activate more specific semantic representations. Sidhu, Vigliocco and Pexman (2020) similarly proposed that iconic words might possess additional and/or more direct links between form and meaning. However, such a mechanism is less likely to explain form typicality effects given the evidence that iconicity and form typicality are distinct constructs, with iconic forms being mostly limited to correspondences between acoustic-phonetic properties of the speech signal and sensory percepts, whereas systematic form-meaning mappings are usually both language-specific and more extensively represented across a vocabulary (Dingemanse et al., 2015). In our view, language processing models that implement Bayesian statistical inference as a prediction mechanism are well-placed to incorporate a priori knowledge about systematic form-meaning mappings represented in the English lexicon, in keeping with a statistical learning framework for language acquisition and representation (Arciuli, 2018; Arciuli & Conway, 2018). Some Bayesian models of speech perception already acknowledge a role for language-specific statistical information represented in phonotactic probabilities—the positional frequency with which phonological segments and sequences of segments occur legally within a language (e.g., Kleinschmidt & Jaeger, 2015; Norris & McQueen, 2008). Similarly, Bayesian models can account for multiple effects in visual lexical decision, reading aloud, and semantic category decision (e.g., Kinoshita, 2015; Norris, 2013), and so that, could be extended to include a priori knowledge about systematic form-meaning mappings represented in the lexicon.

In conclusion, our findings add to the existing evidence for non-arbitrary form-meaning relationships with semantic size in English by demonstrating extensive statistical regularities, that is, form typicality, in a large sample of words of various classes. Furthermore, we show that size form typicality is a more consistent predictor of lexical access in word comprehension and production than semantic size, and that it plays an important role in verbal memory. Future studies might consider exploring non-arbitrary statistical form-meaning relationships with semantic size in other languages.

## Notes

1 The majority of these words were monomorphemic (*N* = 2,142; 73.25%). Restricting the analyses to monomorphemic words resulted in a best fit model with 13 form variables that accounted for a smaller proportion of variance (adjusted *R*^2^ of .167).
